# Impact of Different Time Intervals on the Color Stability of Glass Ionomer Cement and Composite Materials Bonded to Silver Diamine Fluoride: An In Vitro Study

**DOI:** 10.7759/cureus.75711

**Published:** 2024-12-14

**Authors:** Ashwanth Sekar, Kavitha Ramar

**Affiliations:** 1 Pediatric Dentistry, SRM Kattankulathur Dental College, Chennai, IND; 2 Pedodontics and Preventive Dentistry, SRM Kattankulathur Dental College, Chennai, IND

**Keywords:** color stability, composite restoration, dental decay, glass ionomer cement (gic), silver diamine fluoride (sdf)

## Abstract

Objective

This in vitro study evaluated the impact of different time intervals on the color stability of glass ionomer cement (GIC) and composite materials bonded to teeth treated with silver diamine fluoride (SDF). Specifically, the study sought to determine if immediate or delayed application of these restorative materials affects the degree of staining caused by SDF.

Materials and methods

Twenty-eight extracted primary molars with cavitated lesions were randomly divided into four groups, each comprising seven samples. SDF was applied to all samples, followed by either immediate or delayed restoration using GIC or composite materials. Group I received immediate composite restorations, Group II received immediate GIC restorations, Group III received composite restorations after a two-week delay, and Group IV received GIC restorations after a two-week delay. Color measurements were taken at various time points using a digital spectrophotometer, and the color difference (ΔE value) was calculated using the CIELAB color space system.

Results

Composite restorations exhibited better color stability than GIC. Immediate restorations had higher discoloration (ΔE: composite 4.25, GIC 26.95). Delaying restorations by two weeks reduced discoloration (composite 1.66, GIC 10.66), with composites showing minimal color change.

Conclusion

Composite restorations exhibited superior color stability compared to GIC, and delayed restoration reduced SDF-induced staining more effectively than immediate restoration.

## Introduction

Over the past century, the importance of preserving natural tooth structure and promoting biological adaptation in managing dental caries has driven the development and acceptance of treatments that prioritize maximum intervention with minimal invasiveness [1].

Nishino et al. established the concept of using silver diamine fluoride (SDF) to treat tooth cavities due to its strong bactericidal properties in 1969 [2]. Fortunately, SDF is now a low-cost and safe solution that can be applied to arrest the progression of caries [3,4]. SDF has demonstrated encouraging outcomes in various randomized clinical trials and systematic reviews regarding controlling dental caries, especially in primary teeth [5-7] and root surface caries in permanent teeth [8-12].

In a systemic review and meta-analysis of clinical studies on SDF application before restoration in children with caries, Gao et al. (2016) found that it effectively prevented 81% of caries (95% CI 68-89%). According to Gao et al. (2016) and Garg et al. (2019), there were no problems associated with SDF treatment, save for the unappealing black staining of the afflicted lesion [3,4].

The stain is the consequence of SDF reacting with airborne components. After SDF application, discoloration has been attributed to the formation of different silver compounds, including silver phosphate precipitate, silver chloride, and silver thiocyanate (13). Topical SDF application is effective due to its ability to cause dentin sclerosis, its silver nitrate component's germicidal properties, and fluoride's proven ability to remineralize teeth [13]. The methods involve the use of potassium iodide (KI) application to delay discoloration and the use of bleach to reduce discoloration. However, these methods are not advisable because their cosmetic benefits are unclear and they disrupt the function of the SDF.

The durability of the treatment can be understood through the color stability of tooth-colored dental restorations, which is a crucial clinical parameter [14]. In clinical scenarios, a ΔE value less than 3.3 is generally acknowledged as indicative of acceptable color stability. Therefore, any ΔE increase exceeding 3.3 could be characterized as indicating reduced color stability [15]. To conceal the enduring discoloration resulting from SDF applications, it would be beneficial to delve deeper into different tooth-colored restorative materials and the timing of their application after SDF treatment as they present choices. The objectives of this study are as follows: (1) to determine if applying a composite or GIC restoration following SDF application to caries-infected teeth results in reduced staining; Also, (2) to determine if the immediate or delayed application of the restoration is linked with less staining.

## Materials and methods

The study involved using 38% SDF liquid (Kids-e-Dental, India), which was selected based on an optimal concentration from a systematic review. For composite restorations, 37% phosphoric acid etching gel (N-tech, Ivoclar, Switzerland) and dental adhesive (Te-EconomBond, Ivoclar, Switzerland) were applied. An 800 MW/cm² LED polymerization light (Bluephase N MC) was used for light curing, while the composite restoration utilized shade A2 Tetra N-Cream Bulk Fill (Ivoclar, Switzerland). For glass ionomer restorations, type II GIC (SHOFU Dental Corporation, United States) was used. Color measurements were conducted using a Digital Spectrometer (VITA Easyshade® V, Germany).

This was a laboratory study conducted on 28 caries-affected teeth, divided into four groups. Two groups received restorations immediately after the SDF application (composite or GIC), and the other two groups received restorations two weeks after the SDF application. Discoloration was measured at several time points throughout the study. The study was approved by the Institutional Review Board (IRB) of SRM Kattankulathur Dental College and Hospital, SRM University, India (SRMIEC-ST0724-1487).

The sample size of 28 primary molars was determined using G power 3.1.9.7, with 95% power, an effect size of 0.05, and a significance level of 0.05. The teeth were randomly distributed using a random number generator (TextMagic tool). These molars were extracted due to late shedding or orthodontic reasons, and teeth with a prior history of restoration, developmental anomalies, or gross decay were excluded.

The teeth were divided into four experimental groups. Group I received immediate composite restorations, while Group II received immediate GIC restorations. Groups III and IV received delayed composite and GIC restorations, respectively, two weeks after the SDF application. Groups I and II represent current clinical practices, while Groups III and IV represent an alternative schedule. Discoloration was measured at several time points across all groups.

The procedure involved applying SDF to all samples for one to two minutes using a micro brush. Composite restorations in Groups I and III were applied after using 37% phosphoric acid etching gel for 20 seconds, followed by rinsing, drying, adhesive application, and polymerization with light curing. GIC restorations in Groups II and IV used self-cured GIC material. After SDF application, the samples in Groups III and IV were stored in distilled water at 37°C for two weeks before restoration.

Color measurements were taken at standardized time points using a digital spectrophotometer. T1 referred to when the restorations were applied immediately after SDF in Groups I and II, and T2 represented two weeks post-restoration. T3 referred to the time of restoration two weeks after SDF application in Groups III and IV, with T4 marking two weeks post-restoration. The ΔE value, representing color differences, was calculated using the ΔELab equation: ΔE* = [(ΔL*)² + (Δa*)² + (Δb*)²]½ [15]. These values were measured in the CIELAB color space, with L* representing brightness, a* indicating red-green, and b* representing yellow-blue. The measurements were taken three times for each sample at different angles and then averaged to calculate the ΔE values.

The collected data were entered into a Microsoft Excel spreadsheet and subjected to statistical analysis using SPSS software version 20.0, IBM, USA. The intergroup comparison between the GIC and composite immediate and delayed parameters was analyzed using ANOVA, while intragroup comparisons were conducted using the Student's t-test. A p-value of less than or equal to 0.05 was considered statistically significant.

## Results

**Figure 1:**
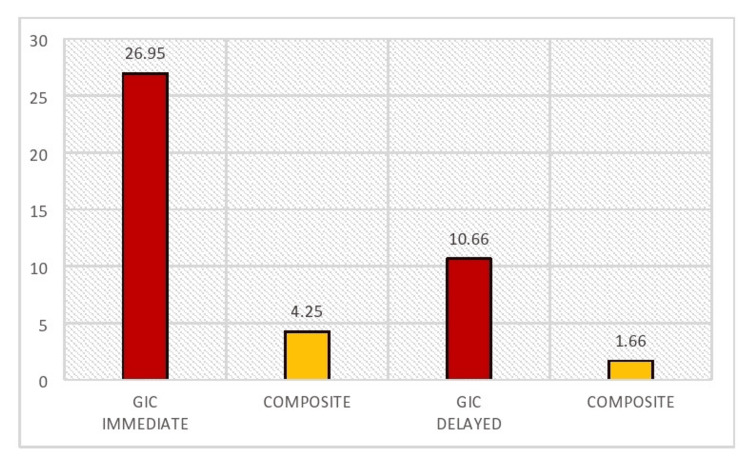
Intergroup comparison between the study groups with respect to ΔE* GIC, glass ionomer cement

The study assessing colorimetric changes in glass ionomer cement (GIC) and composite materials after the application of SDF in 28 samples revealed significant differences between the two materials across both immediate and delayed restoration methods. According to Table 1 and Figure 1, intergroup comparisons using the ANOVA test showed a statistically significant difference (p=0.000) in colorimetric changes between GIC and composite materials in both immediate and delayed restoration groups. In the immediate group, composite materials exhibited much smaller changes (4.25±2.612) compared to GIC (26.95±7.102), and in the delayed group, composite changes (1.66±0.845) were also notably less than those in GIC (10.66±2.741). These findings indicate that composite materials resulted in significantly fewer color changes than GIC in both immediate and delayed scenarios.

**Table 1:** Intergroup comparison between the groups with respect to ΔE* ANOVA test, p≤0.05 GIC, glass ionomer cement

Parameter	Groups	Mean	SD	95% CI		Significance
				Lower	Upper	
Immediate	GIC	26.95	7.102	16.45947	28.92338	0.000
	Composite	4.25	2.612			
Delayed	GIC	10.66	2.741	6.64037	11.36534	0.000
	Composite	1.66	.845			

**Figure 2:**
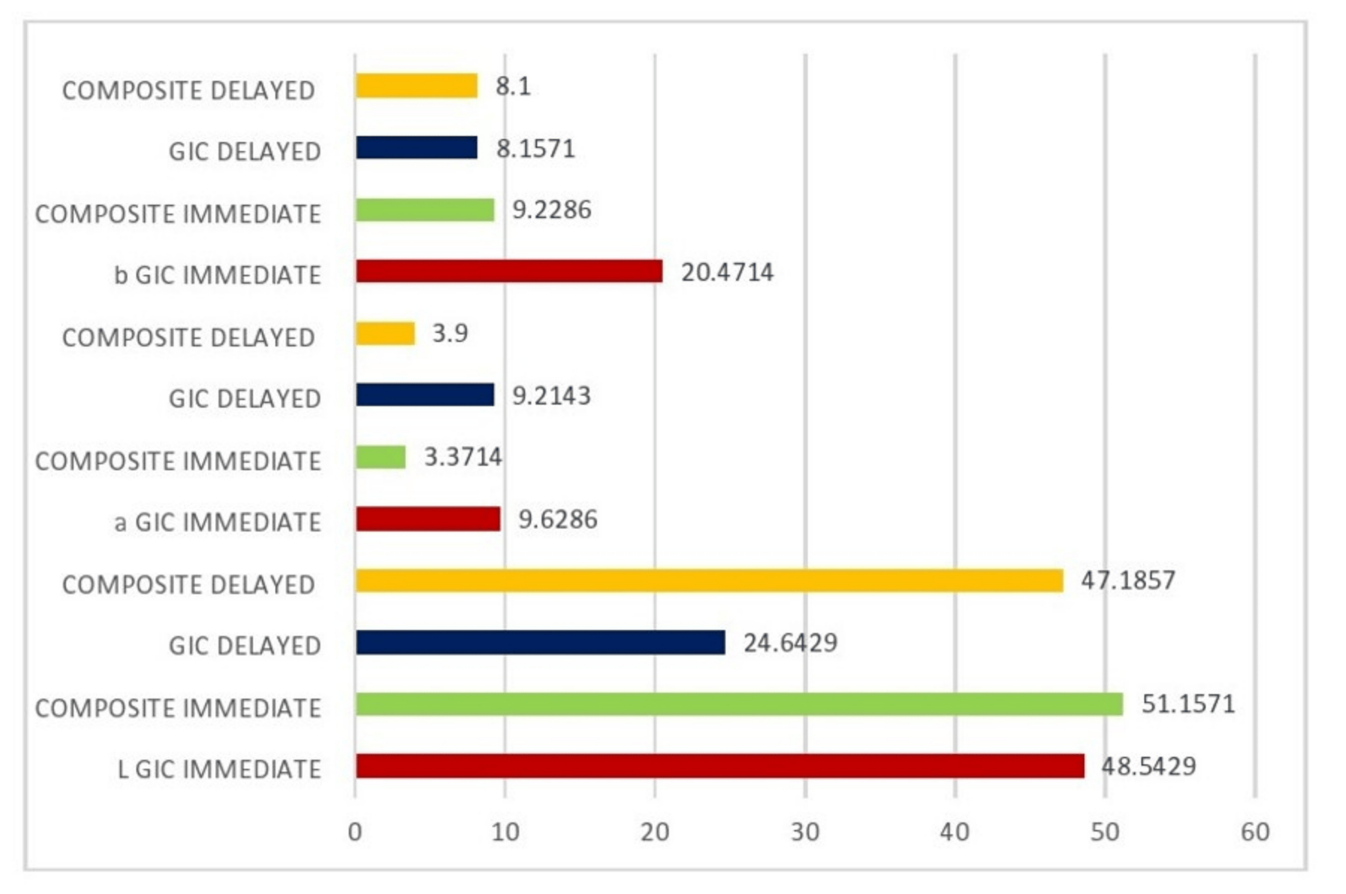
Comparison between the study groups and colorimetric parameters GIC, glass ionomer cement

Table 2 and Figure 2 provide further insight into intergroup comparisons of colorimetric parameters (L, a, and b) using ANOVA, showing a highly significant statistical difference (p<0.005) between GIC and composite groups. Across all parameters, l (lightness), a (red-green axis), and b (yellow-blue axis), composite materials, both in immediate and delayed groups, demonstrated better color stability compared to GIC. The composite groups consistently showed less colorimetric variation, making them superior in terms of maintaining aesthetics.

**Table 2:** Comparison between the study groups and colorimetric parameters ANOVA test, p≤0.05 GIC, glass ionomer cement

Colorimetric parameter	Groups	Mean	SD	95% CI		Significance
				Lower	Upper	
L	GIC Immediate	48.5429	1.99571	46.6971	50.3886	0.001
	Composite Immediate	51.1571	3.85437	47.5924	54.7218	
	GIC delayed	24.6429	4.83351	20.1726	29.1131	
	Composite delayed	47.1857	1.71117	45.6031	48.7683	
A	GIC immediate	9.6286	.49570	9.1701	10.0870	0.002
	Composite immediate	3.3714	.34983	3.0479	3.6950	
	GIC delayed	9.2143	2.06755	7.3021	11.1265	
	Composite delayed	3.9000	.53852	3.4020	4.3980	
B	GIC immediate	20.4714	1.01442	19.5332	21.4096	0.000
	Composite immediate	9.2286	3.16160	6.3046	12.1526	
	GIC delayed	8.1571	1.77844	6.5124	9.8019	
	Composite delayed	8.1000	1.98997	6.2596	9.9404	

**Figure 3:**
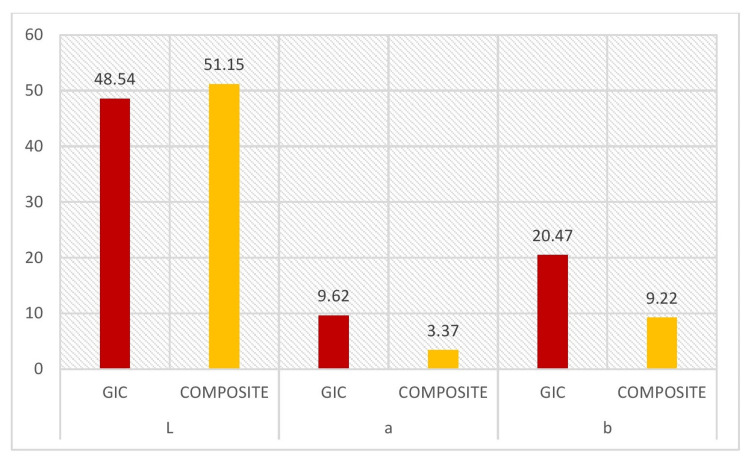
The intragroup comparison between GIC immediate and composite immediate in relation to the colorimetric test GIC, glass ionomer cement

**Figure 4:**
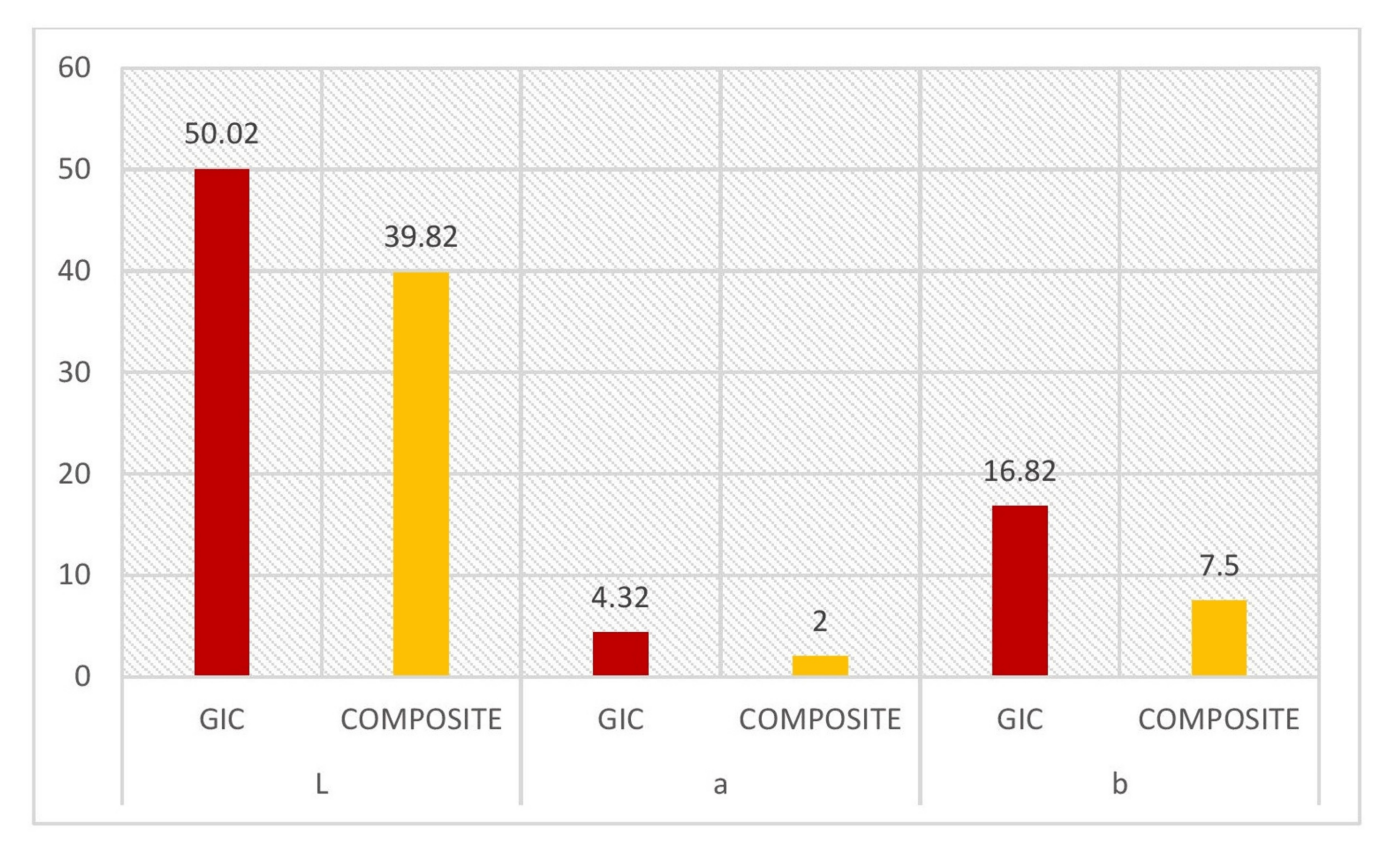
The intragroup comparison between GIC delayed and composite delayed in relation to the colorimetric test GIC, glass ionomer cement

In Table 3 and Figure 3, the intragroup comparison between GIC and composite materials for the immediate restoration group using the Student's T-test revealed no significant difference in the L parameter. However, for the a and b parameters, a significant difference was observed, with the composite group showing better colorimetric performance than the GIC group. Similarly, Table 4 and Figure 4 present the intragroup comparison between GIC and composite materials in the delayed group, where significant differences were found across all colorimetric parameters (L, a, and b), favoring the composite material.

**Table 3:** The intragroup comparison between GIC immediate and composite immediate in relation to the colorimetric test Student's T-test for intragroup comparison, p≤0.05 GIC, glass ionomer cement

Colorimetric parameter	Materials	Mean	SD	95% CI		Significance
				Lower	Upper	
L	GIC	48.54	1.995	-6.188	.960	.137
	Composite	51.15	3.854			
A	GIC	9.62	.495	5.757	6.756	.000
	Composite	3.37	.349			
B	GIC	20.47	1.014	8.508	13.977	.000
	Composite	9.22	3.161			

**Table 4:** The intragroup comparison between GIC delayed and composite delayed in relation to the colorimetric test Student's T-test for intragroup comparison, p≤0.05 GIC, glass ionomer cement

Colorimetric parameter	Materials	Mean	SD	95% CI		Significance
				Lower	Upper	
L	GIC	50.02	4.059	5.33931	15.06069	.001
	Composite	39.82	4.284			
A	GIC	4.32	1.615	.89531	3.76183	.002
	Composite	2.00	.648			
B	GIC	16.82	4.865	5.30999	13.34715	.000
	Composite	7.50	.369			

These results collectively suggest that composite materials offer better colorimetric stability and aesthetic outcomes compared to GIC after the application of SDF, both in immediate and delayed restoration methods. Composite materials demonstrated significantly less color change and greater consistency in colorimetric parameters, making them more effective in preserving aesthetics in clinical restorations.

## Discussion

According to the findings of this in vitro investigation, there were noticeable colour changes in the restorations after SDF treatments. The null hypothesis has been rejected since there was less discoloration observed when the restorative materials were loaded two weeks after SDF treatment as opposed to the immediate loading. It was found that spectrophotometers and the CIE L*a*b* color coding scheme are examples of colorimetric devices that are suitable for objectively detecting color changes that are invisible to the human eye. The numerical data collected from the afflicted areas of the lesion and sound allowed for an effective study of the color changes [16].

The color changes in restorative materials put onto sound dentin and at two-time intervals-immediately and two weeks after SDF administration on demineralized dentin-were measured in this study using a spectrophotometer based on the CIE L*a*b* system. The American Academy of Paediatric Dentistry recommendations suggest that to improve aesthetics, the treated and arrested cavitated caries lesions by SDF may be restored. In this study, a delayed restoration after two weeks of SDF treatment showed superior color stability to cover the dark staining of the SDF-treated demineralized dentin. Timing of restoration loading is an essential element to consider [17].

The results are consistent with a similar study in which the authors suggested postponing the restorations for two weeks to lessen the discoloration produced by SDF. A repair to cover the persistent staining induced by SDF may be postponed for up to a week. The one-week waiting period has drawbacks, including patient compliance, the potential for SDF's anti-caries effect to fade after application, and the function of the oral microbiome. Moreover, poor oral hygiene and eating habits may make it more difficult for SDF to maintain caries arrest [18].

Therefore, it is crucial to get informed permission and to discuss the advantages and disadvantages of using SDF as well as the potential need for restorations when the caries is arrested. Applying SDF two weeks before restorations in pediatric patients raises concerns mostly about potential disruptions to the SDF's anti-caries activity. Furthermore, in the two weeks between the administration of SDF and restoration, it is unknown what effect regular exposure to food, saliva, and dental hygiene products will have on the caries lesion. The treatment recommendations should be modified to include this delay between the administration of SDF and restoration if these laboratory results are confirmed in the field. Another practical issue is that making two appointments is necessary.

This study has limitations due to its in vitro design, which may not support the in vivo settings, such as variations in salivary parameters, a large number of oral microbiomes, and oral environmental variables that might affect the use of restorative therapies and SDF in the management of caries. Moreover, in vitro research may merely rank the materials or methods rather than identify the true way of color stability. As such, care should be used while interpreting the data. When comparing the improvements in discoloration after 7 days, the period when the hard, stained dentin emerged after the application of SDF was not taken into consideration.

Patel et al. found that the black staining appeared within two minutes of the SDF treatment and that its value grew over time regardless of the SDF application duration or concentration (12% or 38%) [19]. Digital photos of the specimens under use must be taken throughout long-term clinical investigations to get proof of color changes that may be taken into account for further study. After two weeks of SDF application, using tooth-colored restorative materials to lessen the unsightly stains may be a sensible alternative treatment strategy. This study might be a useful contribution to SDF research, as the staining potential of SDF has become increasingly apparent. A delay of two weeks after the SDF application to perform the restoration would give ample time for the activity of arresting caries to take place. This approach needs to be explored in patients to understand its effectiveness and acceptance as a treatment modality.

## Conclusions

The study highlights the impact of SDF application timing on the color stability of restorative materials, specifically composite resin and GIC. It was observed that composite restorative materials demonstrated better color stability when SDF application was delayed compared to immediate application. This suggests that the interaction between SDF and composite materials may be less pronounced or mitigated when there is a time gap between the restorative procedure and the application of SDF.

Conversely, GIC showed less favorable color stability in both immediate and delayed SDF application groups, indicating that its interaction with SDF is more pronounced irrespective of timing. This may be due to the inherent chemical properties of GIC, which make it more reactive to the components of SDF. The findings underscore the importance of considering both the type of restorative material and the timing of SDF application to minimize aesthetic compromises, particularly in cases where color stability is a critical clinical outcome.
